# Feasibility of artificial intelligence-based rapid on-site evaluation for the diagnosis of pulmonary disease

**DOI:** 10.3389/fonc.2026.1836366

**Published:** 2026-05-21

**Authors:** Yuxian Chen, Chengcheng Du, Pengchen Gu, Chunhai Li, Hong Meng, Fanlei Kong

**Affiliations:** 1 Department of Radiology, Qilu Hospital of Shandong University, Jinan, China; 2 Shanghai Aitrox Technology Corporation Limited, Shanghai, China

**Keywords:** artificial intelligence, exfoliated cell print, percutaneous core needle biopsy, pulmonary lesions, rapid on-site evaluation

## Abstract

**Introduction:**

This study aimed to investigate the clinical feasibility of artificial intelligence-rapid on-site evaluation (AI-ROSE) based on exfoliated cell blotting from percutaneous puncture biopsy specimens for diagnosing pulmonary lesions and to provide a reference for intraoperative rapid diagnosis.

**Methods:**

A total of 266 patients with pulmonary lesions who underwent computed tomography (CT)-guided percutaneous core needle biopsy between June 11 and November 27, 2024, were enrolled. Exfoliated cell prints from biopsy specimens were stained with Diff-Quik, followed by diagnosis using AI-ROSE. Using the final histopathological diagnosis as the standard, the accuracy, sensitivity, specificity, positive predictive value (PPV), and negative predictive value (NPV) of AI-ROSE and conventional cytological diagnoses were compared. The consistency between the methods and histopathological diagnosis was analyzed. The diagnostic times for AI-ROSE, cytology, and histopathology were recorded and compared. Postoperative complications were documented, and the correlation between lesion characteristics and complications was analyzed.

**Results:**

Compared with histopathological results, AI-ROSE achieved a sensitivity of 95.67%, specificity of 79.31%, diagnostic accuracy of 92.11%, PPV of 94.31%, and NPV of 83.64% in diagnosing pulmonary lesions, with good consistency (κ = 0.764, P < 0.001). No significant difference in the AI-ROSE diagnostic accuracy was observed among the different pathological types (P > 0.05). Conventional cytological diagnosis showed a sensitivity of 87.50%, specificity of 85.71%, and accuracy of 87.10%, with lower consistency and histopathology (κ = 0.665, P < 0.001) than those of AI-ROSE. The mean diagnostic time of AI-ROSE was 254.60 ± 13.88 s, which was significantly shorter than that of cytology (1.48 ± 0.86 days) and histopathology (2.37 ± 1.42 days). The overall incidence of postoperative complications was 22.93%, including pneumothorax (12.78 %) and needle tract bleeding/mild hemoptysis (9.77%), with no fatal complications. A smaller nodule volume was associated with a higher risk of puncture bleeding (P = 0.004), whereas lesion size and puncture path length were not significantly correlated with pneumothorax risk (P > 0.05).

**Discussion:**

AI-ROSE enables rapid intraoperative diagnosis of pulmonary lesions in percutaneous biopsy with high diagnostic performance and consistency with histopathological results, demonstrating its favorable value for clinical application and popularization.

## 1 Introduction

Lung cancer is the most prevalent cancer and a leading cause of cancer-related death worldwide (1, 2). Many lung-occupying lesions on chest computed tomography (CT) scans are indicative of lung cancer. However, by the time of diagnosis, approximately 70% of lung cancer cases are inoperable, highlighting the need for early detection and lesion characterization (3). Although other diseases, such as tuberculosis and lung infections, must be considered in the differential diagnosis, CT-guided percutaneous core needle biopsy is crucial for diagnosing lung cancer (4–6). However, the sampling success largely depends on the operator’s experience. Additionally, patients must wait for histopathology reports to confirm the results, which can be time-consuming. If tumor tissue puncture fails, a second biopsy may be necessary, increasing discomfort, time, and expense.

The use of artificial intelligence-rapid on-site evaluation (AI-ROSE) in pulmonary diagnosis involves acquiring diseased tissue using methods such as percutaneous lung biopsy, transbronchial biopsy, and alternative manipulations (7–9). This process includes immediate printing and staining of exfoliated cells, allowing a swift assessment of the biopsy success and specimen adequacy, facilitating a timely preliminary diagnosis. AI-ROSE has been used in endobronchial ultrasound-guided fine-needle aspiration biopsy, demonstrating enhanced diagnostic efficacy, reduced puncture frequency, lower complication rates, and lower costs (10–12). This study aimed to investigate the feasibility of AI-ROSE in diagnosing pulmonary lesions using the exfoliated cell print of a percutaneous core needle biopsy specimen.

## 2 Methods

### 2.1 Patients

This study included 266 patients with pulmonary lesions who underwent CT-guided biopsy at our institution between June and November 2024. The inclusion criteria were as follows: (1) CT scans revealing single or multiple nodules or masses with ineffective anti-infective treatment; (2) imaging suggesting malignancy, with follow-up examinations indicating lesion enlargement; (3) a clarified pathological type necessitating genetic testing; (4) recurrence following antitumor treatment, requiring clarification of drug resistance mechanisms; and (5) no biopsy contraindications. The exclusion criteria were as follows: (1) severe coagulopathy or cardiopulmonary dysfunction, (2) inability to cooperate due to severe coughing, mental disorders, or other factors, (3) significant bleeding risk during biopsy, and (4) incomplete patient information. This study was approved by our Institutional Review Board, which waived the requirement for informed consent. All patients provided written informed consent before undergoing the procedure.

### 2.2 Principle of AI-ROSE

**Figure 1:**
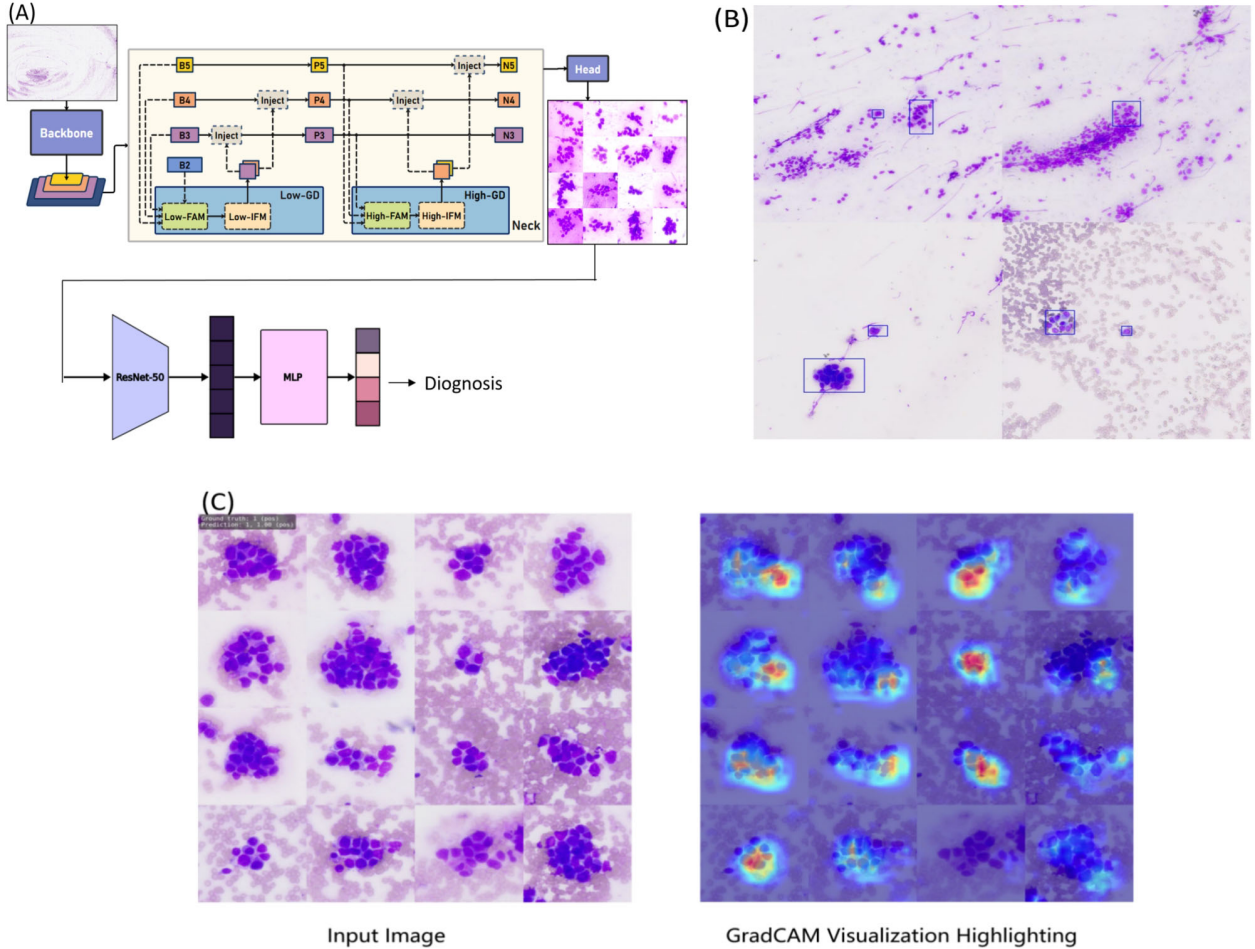
A multi-step attentional learning-based model for rapid on-site diagnosis in pathological cytology. **(A)** algorithm flow chart; **(B)** Object Detection Phase; **(C)** input image and highlighting of the diagnostic basis. Inject, information injection module; FAM, feature alignment module; IFM, information fusion module; GD, gather-and-distribute module; MLP, multilayer perceptron.

We proposed a two-stage AI-ROSE model with a multistep attention mechanism (**Figure 1A**). This AI system evaluates cytological slide images. The architecture includes an object-detection model followed by a classification model. The object detection model uses a multipath information fusion network architecture, with ViT-B/16 serving as the backbone, to distinguish between normal and lesional tissues, detect abnormal cells. To enhance the generalization ability of the backbone, MoCoV2 was used for self-supervised learning on unlabeled and weakly labeled datasets. To process the whole-slide images, a sliding-window mechanism was used to crop patches of size 1024 × 1024, which were sequentially fed into the object detection model to identify the bounding boxes around the abnormal cells (**Figure 1B**). Using the detected abnormal cell bounding boxes, we extracted the regions suspected to contain the abnormalities. Multiple suspected regions were cropped and stitched into a single 2048 × 2048 mosaic image, providing a centralized representation of the suspected abnormal areas (**Figure 1C**). This mosaic image was subsequently fed into the classification model to determine the malignancy or benignancy of the entire slide. The classification model used a lightweight ResNet50 as the backbone, followed by a multilayer perceptron for the classification outputs. This design leveraged the synergy between advanced feature extraction and focused classification, ensuring robust and accurate predictions for whole-slide pathological analysis.

### 2.3 Procedure of biopsy

Before surgery, the clinician informed the patients about the purpose and precautions of the puncture procedure. All patients underwent a series of preoperative evaluations, including routine blood tests, coagulation tests, infection screening, pulmonary function tests, and electrocardiograms, to assess cardiopulmonary function and cooperative ability. Chest-enhanced CT was performed to identify the blood supply characteristics of the lesion and adjacent structures. Based on these evaluations, clinicians determined the optimal puncture strategy, including the point of needle insertion, direction of needle insertion, and depth. A Siemens third-generation dual-source CT was used. The patient was optimally positioned according to the puncture path, and the area was disinfected, covered with sterile towels, and anesthetized with 2% lidocaine. Axial chest scanning was performed using the following parameters: tube voltage, 120 kV; tube current, 140 mA; layer thickness, 5 mm; and layer spacing, 5 mm. A biopsy was performed by an interventionalist with over 10 years of experience using an 18G cutting biopsy needle. Care was taken to ensure that at least one tissue sample was obtained. The exfoliated cells were imprinted onto the specimens. The specimens were fixed in formalin and sent for examination. After the puncture, chest CT was performed to observe any complications, such as pneumothorax, and symptomatic treatment was provided when necessary. Patients were instructed to rest in bed for 4–6 h post-procedure to avoid any strenuous activities.

### 2.4 Procedure of AI-ROSE

After biopsy, the tissue was carefully extracted using a disposable 5 mL syringe needle and evenly smeared onto a cytology slide in a circular motion from the inside out, creating a moderate thickness of approximately 1–1.5 cm in diameter for AI-ROSE analysis. After smearing, the remaining tissue was carefully placed in a pathology vial and sent for examination. At the surgical site, AI-ROSE cytological smears were rapidly stained with Diff-Quik dye by immersing the slides in Diff A solution for 30–50 s and rinsing in a phosphate buffer staining bath to remove excess Diff A solution. The slides were dried and immersed in Diff B solution for 5–8 s to complete the staining. After rinsing the slides in water to remove any residual dye and drying them with absorbent paper, the slides were analyzed under a microscope.

### 2.5 Diagnostic criteria

Diagnosis was based on histopathological findings of the biopsy, which is the gold standard. Lesions that showed malignancy were considered cancerous. Diagnostic criteria for benign lesions: Patients whose biopsy histopathology results show benign lesions are confirmed if the lesions shrink, disappear, or remain unchanged for more than 1 year. All cytology results were independently reviewed by two senior cytologists with ≥10 years of experience. Discrepant cases were further evaluated by the chief cytopathologist to reach a consensus.

### 2.6 Statistical analysis

All statistical analyses were performed using the SPSS software (version 27; SPSS, Inc.). The counting data are expressed as percentages (%), with comparison by χ2 inspection. Measurement data were expressed as x ± s, and a t-test was used for comparison. The correlations among AI-ROSE, cytology, and final pathological results were assessed using the Kappa statistic. Accuracy, sensitivity, and specificity were calculated. Statistically significance was set at P < 0.05.

## 3 Results

### 3.1 Patients

**Figure 2:**
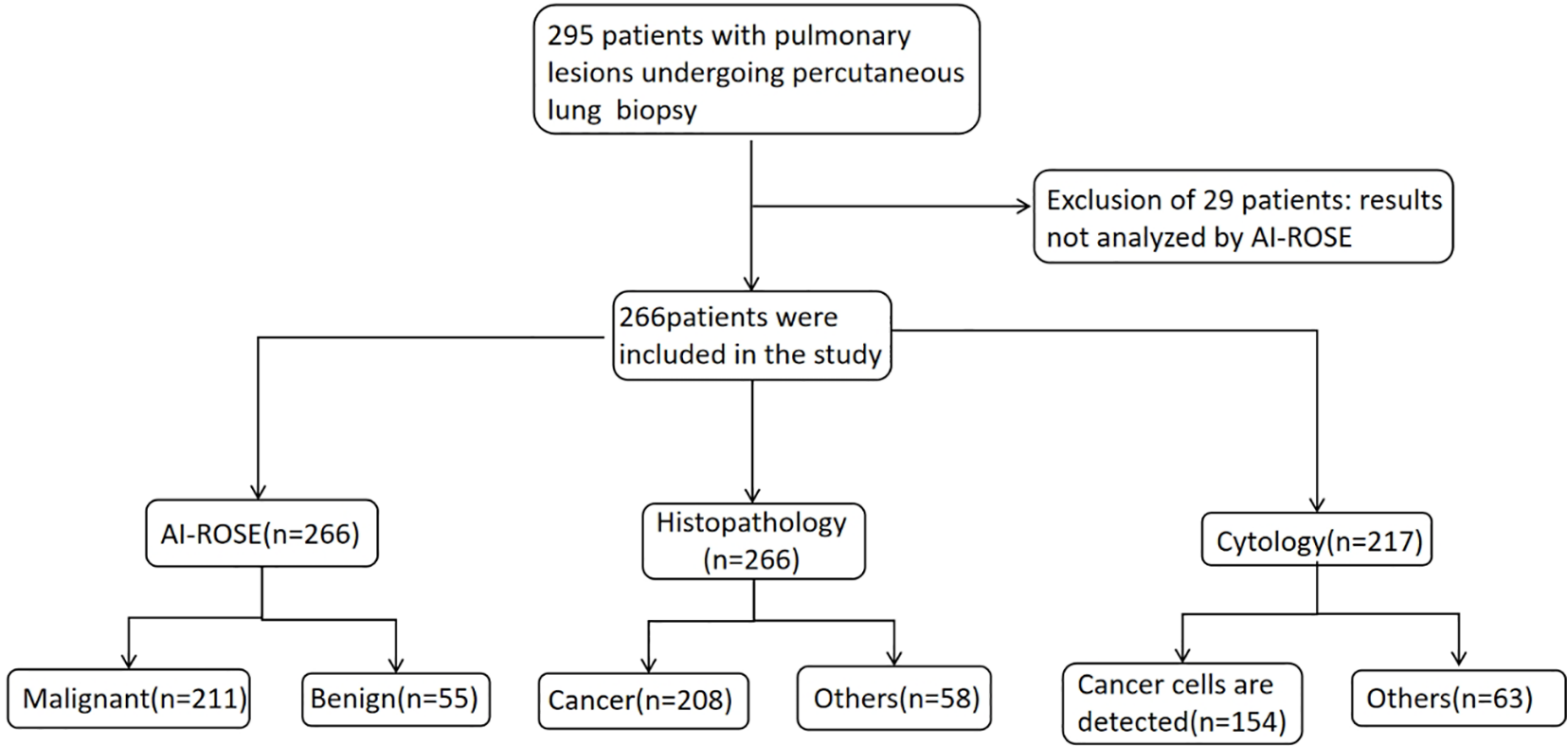
Flowchart for the selection and diagnosis of the study population.

In this study, 295 patients were initially enrolled, of whom 29 were excluded and 266 were included, as illustrated in **Figure 2**. The cohort comprised 156 men and 110 women, aged 26–88 years, with a mean age of 66.38 ± 10.97 years. Lesion sizes varied from 7.6 to 163.6 mm, with a mean size of 39.54 ± 24.29 mm. Furthermore, the distance of the lesions from the pleura ranged from 0 to 51 mm, with a mean distance of 6.70 ± 9.34 mm. Most lesions (65.04%) were located in the upper lobe. The patient characteristics are shown in **Table 1**.

**Table 1:** Characteristics and clinical presentation of patients undergoing percutaneous lung biopsy (n = 266).

Characteristics	No. of patients (%)
Age (years).mean ± SD	66.38 ± 10.97
Gender	
male	156(58.65%)
female	110(41.35%)
Site of lesions	
upper lobe(left)	49(30.83%)
lower lobe(left)	43(16.17%)
upper lobe(right)	91(34.21%)
middle lobe(right)	4(1.50%)
lower lobe(right)	46(17.29%)
Size of lesions(mm).mean ± SD	39.54 ± 24.29
Distance of lesion from pleura(mm).mean ± SD	6.70 ± 9.34

**Figure 3:**
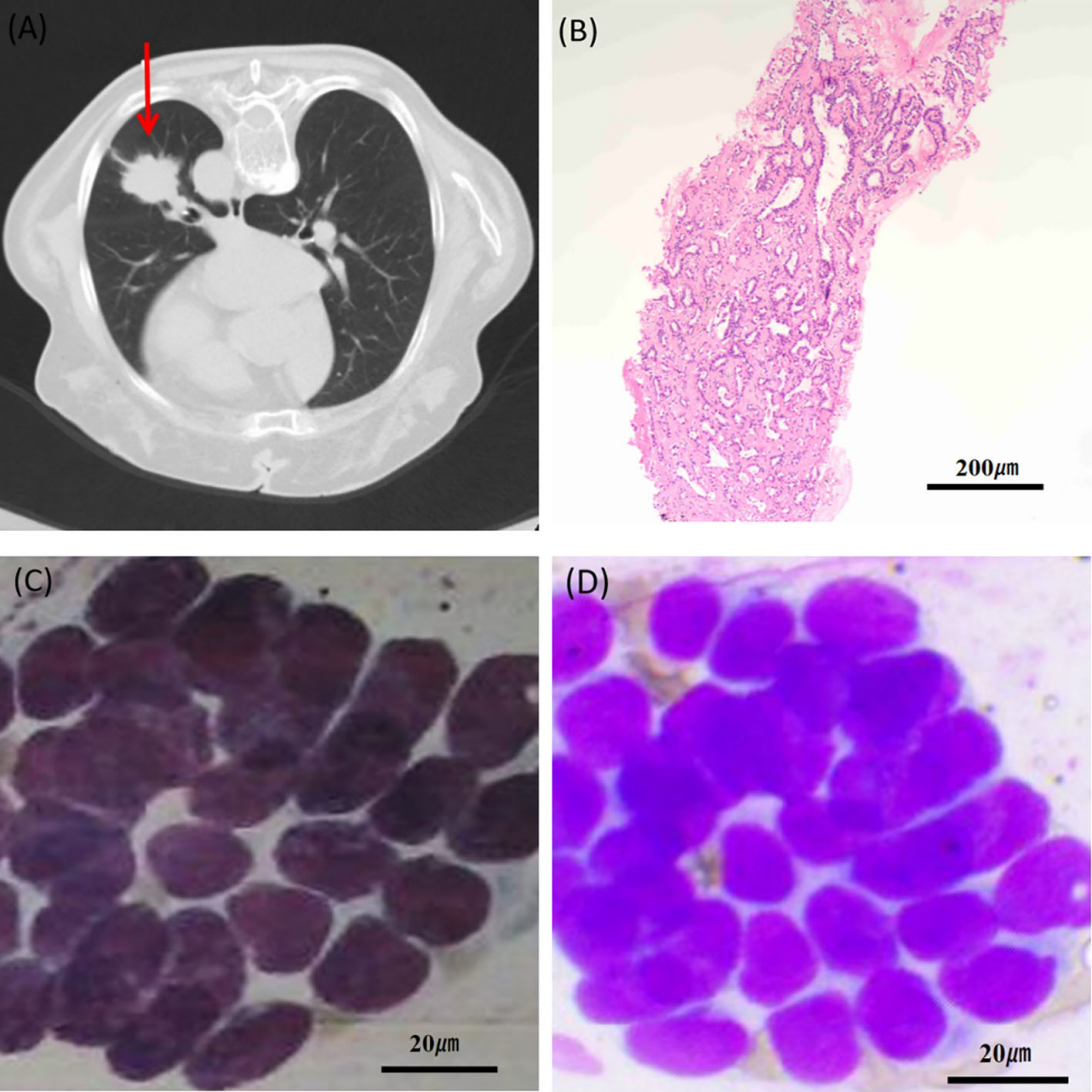
A 72-year-old female patient who underwent CT-guided percutaneous core-needle biopsy. **(A)** CT image of the chest showed a lesion in left lung measuring 48.5 mm (red arrow); **(B)** histopathology showed adenocarcinoma (×100); **(C)** cancer cells detected by cytology (×400); **(D)** AI-ROSE shows suspicious malignant cells (×400). AI-ROSE, Artificial Intelligence-Rapid On-Site Evaluation.

All patients underwent AI-ROSE; 211 and 55 patients exhibited malignant and benign outcomes, respectively. Subsequently, all patients underwent histopathological examination, and the results are presented in **Table 2**. Among these malignancies, 18 patients with identified as metastatic cancers from various primary origins: 1 with lung adenocarcinoma metastasis, 3 with hepatocellular carcinoma metastasis, 1 with breast cancer metastasis, 1 with endometrial carcinoma metastasis, 1 with gestational trophoblastic tumor metastasis, 1 with adrenal sebaceous gland carcinoma metastasis, 5 with gastrointestinal tract tumor metastasis, 1 with epithelial tumor metastasis, and 4 with histiocytic sarcoma metastasis. Unclassified carcinomas lack a specific pathological type on histopathological examination. Benign pathologies include various conditions, such as pulmonary tuberculosis, inflammation, and fibrous hyperplasia. Additionally, 217 of the 266 patients underwent cytological examination, and the findings are presented in **Figure 3**.

**Table 2:** Histopathologic results in patients.

Histopathologic results	No. of patients (%)
Adenocarcinoma	142(53.38%)
adenocarcinoma in situ	10(3.76%)
invasive adenocarcinoma	122(45.86%)
mucinous adenocarcinoma	10(3.76%)
Squamous cell carcinoma	28(10.53%)
Small-cell carcinoma	11(4.14%)
Large cell carcinoma	2(0.75%)
Metastatic carcinoma	18(6.77%)
Unclassified carcinoma	7(2.63%)
Benign pathology	58(21.80%)

In 21 patients, AI-ROSE results differed from the final histopathological diagnoses: 5 adenocarcinomas, 1 squamous carcinoma, 3 metastases, and 12 benign. No significant difference was observed in the diagnostic accuracy across the pathological types (P > 0.05). AI-ROSE had 12 false positives, resulting in a positive predictive value (PPV) of 94.31%, and 9 false negatives, yielding a negative predictive value (NPV) of 83.64%. The sensitivity, specificity, and diagnostic accuracy of AI-ROSE for peripheral lung lesions were 95.67%, 79.31%, and 92.11%, respectively (**Table 3**). Furthermore, AI-ROSE demonstrated a high level of consistency with histopathological results (κ = 0.764, P < 0.001). In contrast, cytology revealed 7 false positives with a PPV of 95.45% and 21 false negatives with an NPV of 66.67%. The sensitivity, specificity, and diagnostic accuracy of cytology for peripheral lung lesions were 87.50%, 85.71%, and 87.10%, respectively (**Table 4**). The cytological results were consistent with the histopathological results (κ = 0.665, P < 0.001).

**Table 3:** Comparison of AI-ROSE results with histopathology results.

AI-ROSE results	Histopathologic results		
	Malignant	Benign	Total
Malignant	199	12	211
Benign	9	46	55
Total	208	58	266

Sensitivity = 95.67% (199/208 cases), specificity =79.31% (46/58 cases), and diagnostic accuracy = 92.11% (139/149 cases).

AI-ROSE, Artificial Intelligence-Rapid On-Site Evaluation.

**Table 4:** Comparison of cytology results with histopathology results.

Cytology results	Histopathologic results		
	Malignant	Benign	Total
Malignant	147	7	154
Benign	21	42	63
Total	168	49	217

Sensitivity = 87.50% (147/168 cases), specificity = 85.71% (42/49 cases), and diagnostic accuracy = 87.10% (189/217 cases).

The AI-ROSE and cytological results differed in 30 patients. The concordance rate between AI-ROSE and cytological diagnosis was 86.18%. Of these, 16 had negative cytology results but positive AI-ROSE and histopathology diagnoses, and 4 had positive cytology results but negative AI-ROSE and histopathological diagnoses. Two patients had negative AI-ROSE but positive cytology and histopathology results, and eight patients had positive AI-ROSE but negative cytology and histopathology results. Thirty-eight patients had AI-ROSE or cytology results inconsistent with the final histopathology results: 10 with AI-ROSE results, 20 with cytology results, and 8 with both inconsistent with histopathology.

The average times for AI-ROSE, histopathology, and cytology were 254.60 ± 13.88 s, 2.37 ± 1.42 days, and 1.48 ± 0.86 days, respectively (**Table 5**).

**Table 5:** Time required for different diagnostic methods.

Methods	Time (second or day)
AI-ROSE	254.60 ± 13.88^a^
Histopathology	2.37 ± 1.42^b^
Cytology	1.48 ± 0.86^b^

AI-ROSE, Artificial Intelligence-Rapid On-Site Evaluation; a: second; b: day.

### 3.2 Complications

Complications were classified using the standardized Society of Interventional Radiology (SIR) grading system (13). Patients who died within 30 days of surgery were classified as SIR F. Major complications were defined as events leading to severe morbidity and disability, equivalent to SIR C–E. This included patients who required blood transfusions or interventional drainage procedures. All other complications were considered to be minor.

All 266 patients underwent a single biopsy, of whom 147 underwent 1 puncture and 2 underwent 2 punctures due to poor respiratory cooperation. The number of CT scans performed during the biopsy ranged from 3 to 21, with a mean of 6.97 ± 2.51. Sixty-one (22.93%) patients experienced post-biopsy complications. Thirty-four (12.78%) patients developed pneumothorax postoperatively, with 7 (2.63%) required closed-chest drainage. Twenty-six patients experienced bleeding from the needle tract or mild hemoptysis, most of whom required no special treatment, and 8 patients were treated with hemostatic drugs or thrombin. Four patients developed postoperative fever and were treated with antibiotics. Four patients developed chest pain and were treated with analgesics (**Table 6**). In our study, the statistical results showed that smaller nodules had no increased pneumothorax or drainage risk, but had an increased risk of bleeding (P = 0.004). Lesions >6 cm had a low risk of puncture bleeding (P = 0.079). No significant difference was observed between the puncture path length and complication rate.

**Table 6:** Complications after CT-guided percutaneous lung biopsy.

Characteristic		No.of patients (%)
Number of punctures		1.01 ± 0.09
Number of needle entries		6.97 ± 2.51
Pneumothorax	A/B	27 (10.15%)
	C	7 (2.63%)
Hemorrhage	A/B	26(9.77%)
Fever	A/B	4(1.50%)
chest pain	A/B	4(1.50%)

## 4 Discussion

Owing to the increased lung nodule detection and rising incidence of lung cancer, there is a growing clinical need for further classification and detailed biosignature analysis (14). CT-guided lung biopsy is commonly used to determine lung nodule nature, with diagnostic accuracy being key in studies (15, 16). Given the intricate pathological image features, diverse disease manifestations, and stringent diagnostic criteria associated with lung lesions, we developed our algorithm design mechanism by addressing the challenges of AI model interpretability, generalization, multiscale data handling, timely model inference, and imbalanced distribution of cell types. Our approach integrates a Vision Transformer and multistep attention mechanism into the model design. The high-capacity transformer enhances feature extraction by capturing nuanced differences and intricate patterns in the pathology images. At the data level, we used self-supervised learning, multiple data augmentation techniques, and robust learning under label noise, using unlabeled and weakly labeled data to improve the model performance. Numerous studies (17–20) have confirmed that AI-ROSE enhances lung biopsy accuracy, reduces the number of punctures, and improves specimen quality. Consequently, integrating CT-guided biopsy with AI-ROSE is crucial for optimizing pathological specimen sampling, augmenting biopsy accuracy, and aiding in lung cancer diagnosis and staging.

AI-ROSE is more prevalent in respiratory endoscopy with biopsy procedures than in CT-guided percutaneous lung biopsy (9, 11, 21). Among the various rapid staining methods used in AI-ROSE, Diff-Quik dye is the most commonly used and is endorsed by the World Health Organization (22). AI-ROSE, a rapid cell smear staining technique, lacks histomorphological detail, limiting its ability to differentiate various lung cancer types. Consequently, it is primarily used for preliminary benign and malignant tumor discrimination. Our study demonstrated that AI-ROSE exhibited high sensitivity (95.67%) and diagnostic accuracy (92.11%), which aligns with the findings of previous studies (23, 24). Positive AI-ROSE results align with rapid on-site assessment and definitive histopathological evaluation, aiding prompt therapeutic decision making. However, the low NPV of 83.64% suggests that a negative AI-ROSE result may indicate suboptimal biopsy sampling, requiring biopsy needle adjustments. If multiple biopsies yield negative AI-ROSE results, awaiting follow-up histopathological outcomes can prevent overtreatment. AI-ROSE should be positioned as an adjunctive intra-procedural tool for rapid evaluation of sample adequacy and preliminary diagnosis, assisting clinicians in real-time decision-making during CT-guided percutaneous lung biopsy, rather than serving as a definitive diagnostic modality. Furthermore, if AI-ROSE detects tumor cells, given its high PPV, the feasibility of tissue retention for direct genetic testing should be explored to expedite diagnosis, reduce the waiting time for histopathological examination, and minimize tissue consumption, which would otherwise be necessary for immunohistochemical analyses. However, the practicality and efficacy of this strategy warrant further investigation in subsequent studies.

Compared to cytology and histopathology, AI-ROSE significantly reduced the diagnostic time (P < 0.001). In analyzing the concordance between AI-ROSE and histopathology results, the inconsistency rate was 6.77% (18/266), with a κ-value of 0.764, indicating a high level of agreement. In contrast, 10.53% (28/266) of cytology results were inconsistent with histopathology results, with a κ-value of 0.665, demonstrating slightly lower consistency compared to AI-ROSE versus histopathology. Furthermore, this study analyzed the diagnostic accuracy of AI-ROSE across different histopathological types and observed no significant differences. This finding suggests that AI-ROSE does not exhibit a significant diagnostic preference for identifying various histopathological types. These findings underscore the substantial advantages of AI-ROSE in improving diagnostic efficiency and confirm its potential value as an auxiliary diagnostic tool. These findings provide robust support for future applications in pathological diagnosis.

As a rapid staining diagnostic method for cell smears, AI-ROSE exhibits high diagnostic accuracy and reduces time requirements, making it a potential alternative to cytology to a certain extent. Additionally, in this study, both AI-ROSE and cytology results were inconsistent with the histopathological results in eight patients. Among these, three patients had histopathologically benign, while the others had malignant; five patients had histopathologically malignant, while the others had benign. This inconsistency may be due to the presence of malignant cells on the slide as a result of rubbing some of the pathological cells without puncturing the tissue during biopsy, or it may be due to the stimulation of inflammatory factors or mediators that cause the cells to degenerate and thus morphologically resemble malignant cells. This inconsistency could be due to the absence of viable cells or cell detachment from the smears. This may account for the low NPV of AI-ROSE, suggesting that negative AI-ROSE results should be histopathologically confirmed.

In this study, 29 patients were excluded because of unsatisfactory AI-ROSE smear results. These issues could be attributed to technician handling problems or smear-related issues, such as missing specimens or smears that were excessively thick or uneven in thickness. In addition, when the smear area is too small, additional identification points can be added to improve the scanning clarity. Consequently, it is imperative that the operator undergoes a brief training period before the procedure with the assistance of a cytologist, if necessary. To prevent cell overlap, which may hinder identification, the smear area can be enlarged, and the smear thickness can be reduced. Furthermore, if necessary, the number of smears can be increased to ensure the accuracy of the results. Although AI-ROSE requires time for staining, preparation, and interpretation, this time can be significantly reduced as the operator gains proficiency (25).

None of the patients experienced serious life-threatening complications, and the complication rate was comparable to that reported in previous studies (26, 27). Postoperative pneumothorax and bleeding were the most frequently encountered complications, most of which were minor complications. The relationship between nodule size and pulmonary hemorrhage is consistent with the findings of a previous study (28). However, no significant correlation was observed between the occurrence of postoperative pneumothorax and the size, depth, or number of needle insertions in the lesion, which may contradict the findings of earlier reports (29).

## 5 Conclusions

Our results demonstrate that AI-ROSE presents promising clinical utility in the diagnosis of pulmonary lesions. As an intraoperative auxiliary tool, AI-ROSE allows for rapid assessment of sample adequacy and preliminary differentiation between benign and malignant lesions, effectively shortens diagnostic time, reduces the risk of overtreatment, and its high sensitivity contributes to timely therapeutic intervention for patients with lung cancer. Nevertheless, this study has the limitations of a relatively small sample size and single-center design. Further large-sample and multicenter clinical studies will be conducted for subsequent verification.

## Data Availability

The raw data supporting the conclusions of this article will be made available by the authors, without undue reservation.
